# Supplementary material for: Pericyte-to-Endothelial Cell Communication via Tunneling Nanotubes Is Disrupted by a Diol of Docosahexaenoic Acid
Source: Cells. 2024 Aug 26;13(17):1429. doi: 10.3390/cells13171429 (PMC11394577; doi:10.3390/cells13171429)
Supplement: Supplementary file 1 [file cells-13-01429-s001.zip › Figure S1.pdf]

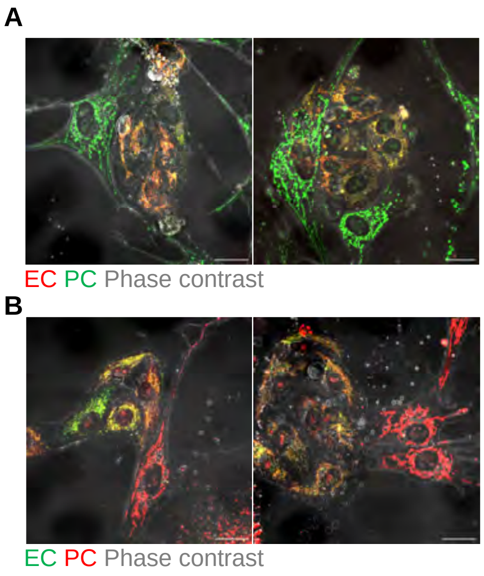

Figure S1. Mitochondrial transfer from pericytes to respiration deficient endothelial cells. (**A**) Mitochondrial transfer from pericytes (MitoTracker Green) to endothelial cells (MitoTracker Red); bar = 20 µm. (**B**) Lack of mitochondrial transfer from pericytes (MitoTracker Red) to endothelial cells (MitoTracker Green). Each panel shows confocal images together with superimposed phase contrast images. Representative images from 3 independent cell batches.
